# eXframe: reusable framework for storage, analysis and visualization of genomics experiments

**DOI:** 10.1186/1471-2105-12-452

**Published:** 2011-11-21

**Authors:** Amit U Sinha, Emily Merrill, Scott A Armstrong, Tim W Clark, Sudeshna Das

**Affiliations:** 1 Department of Pediatric Oncology, Dana-Farber Cancer Institute and Harvard Medical School, Boston, MA 02115, USA; 2 MassGeneral Institute for Neurodegenerative Disease, Massachusetts General Hospital, Cambridge, MA 02139, USA; 3 Department of Neurology, Harvard Medical School, Boston, MA 02115, USA

## Abstract

**Background:**

Genome-wide experiments are routinely conducted to measure gene expression, DNA-protein interactions and epigenetic status. Structured metadata for these experiments is imperative for a complete understanding of experimental conditions, to enable consistent data processing and to allow retrieval, comparison, and integration of experimental results. Even though several repositories have been developed for genomics data, only a few provide annotation of samples and assays using controlled vocabularies. Moreover, many of them are tailored for a single type of technology or measurement and do not support the integration of multiple data types.

**Results:**

We have developed eXframe - a reusable web-based framework for genomics experiments that provides 1) the ability to publish structured data compliant with accepted standards 2) support for multiple data types including microarrays and next generation sequencing 3) query, analysis and visualization integration tools (enabled by consistent processing of the raw data and annotation of samples) and is available as open-source software. We present two case studies where this software is currently being used to build repositories of genomics experiments - one contains data from hematopoietic stem cells and another from Parkinson's disease patients.

**Conclusion:**

The web-based framework eXframe offers structured annotation of experiments as well as uniform processing and storage of molecular data from microarray and next generation sequencing platforms. The framework allows users to query and integrate information across species, technologies, measurement types and experimental conditions. Our framework is reusable and freely modifiable - other groups or institutions can deploy their own custom web-based repositories based on this software. It is interoperable with the most important data formats in this domain. We hope that other groups will not only use eXframe, but also contribute their own useful modifications.

## Background

In the past two decades, numerous repositories have been developed for data management and analysis of genomics studies. The largest and most notable are the public repositories Gene Expression Omnibus [1] and ArrayExpress [2] which store data from variety of different platforms, but allow users to query gene expression only. There are a few efforts to archive the raw data from next generation sequencing runs [3]. However most genomics repositories are still limited to microarrays - examples include the Stanford Microarray Database [4], mAdb [5], Genopolis [6], MiMiR [7] and several others which are compared in a useful review by Gardiner-Garden and Littlejohn [8].

Most of these microarray databases follow the **M**inimum **I**nformation **A**bout a **M**icroarray **E**xperiment (MIAME) standard [9] that specifies the minimum required information needed to enable the interpretation of the results of the experiment. However, they often have heterogeneous sample annotation and use free text rather than a controlled vocabulary, making it difficult to perform integrative meta-analysis across experiments. Several repositories were developed to specifically address this issue, including M2DB - a microarray meta-analysis database of over 10,000 experiments annotated with disease states and organism parts with terms from controlled vocabularies [10]; Oncomine - a web-based data management and mining platform for cancer datasets [11]; GCOD - GeneChip Oncology Database -which has curated human cancer datasets [12] and Genevestigator, which provides annotation on variety of biological contexts [13].

Although structured annotation of samples has allowed researchers to query expression across biological contexts, the actual application of these systems is limited to expression data. To accommodate other types of genomics data (for example from ChIP-Seq or RNA-Seq assays), standardized metadata on experimental design, measurement type and assay technology need to be captured. The ISA software suite (which consists of the ISA-Tab format and supporting tools) was the first successful effort devised to annotate studies with heterogeneous high-throughput assays using standard ontologies [14,15]. While the ISA infrastructure offers significant improvements in the structured annotation of diverse assays, as a metadata format/store, it does not of course provide tools for processing, analysis or visualization of data.

Further, and very importantly, most databases are not available as open-source software to allow local installation and/or customization. This has led to inefficiencies, duplication of effort and creation of numerous databases. Swetrz et al. reviewed a dozen of these genomics databases for maintainability, extensibility and interoperability [16]. Only a few were found to be configurable and for most, the software wasn't readily available for reuse. In reaction to these findings, MOLGENIS was developed as a local experimental genomics database [16,17]; however, it isn't designed or optimized for integrative analysis.

We have developed eXframe, a reusable framework that addresses the issues of standardized annotation, multiple data types and analysis tools in a single platform. Our framework allows storage of gene expression, histone modification and transcription factor binding data from both microarrays and next generation sequencing technologies. The samples and assays are annotated with controlled vocabularies/ontologies and all data is processed and stored in a consistent way. This enables queries across species, experimental conditions and assay types, thus allowing the researcher to compare their data with others. The software is currently being used for two repositories, one containing hematopoietic stem cell data and the other Parkinson's disease patients' data.

## Implementation

In this section we describe the implementation of eXframe and its various components.

### Framework

Web-based systems support ease of distribution, platform independence and scalable architecture. We implemented our system as a web-accessible database built on the LAMP (Linux, Apache, MySQL and PHP) technology stack. All components are available under open-source software license. We leverage the added convenience, power, and extensibility of a widely used open source content management system and social networking tool, Drupal [18]. Drupal is built on the PHP web scripting language, and its persistence store is a MySQL database. Drupal has a large developer community, allows ready customization and is highly scalable.

Several basic modules, such as the user login system, a caching module for fast access of pages, and SOLR [19] based search are pre-packaged with Drupal. It also has a large number of contributed modules that are easily integrated, thus speeding up the development process. Browsing, searching, and filtering capabilities are provided as part of the general Drupal framework.

Drupal also allows granular permissions and security based on user roles and group memberships. We used the granular permissions capability to implement flexible data publication. Users of our system can choose to publish just the experiment metadata and keep the raw or processed data hidden. The experiment metadata allows users to be aware of an experiment that has been performed by another user of the repository, and can thus foster collaboration while still protecting pre-publication data. The raw or processed data can be made public at a later stage when it has been accepted for publication.

**Figure 1:**
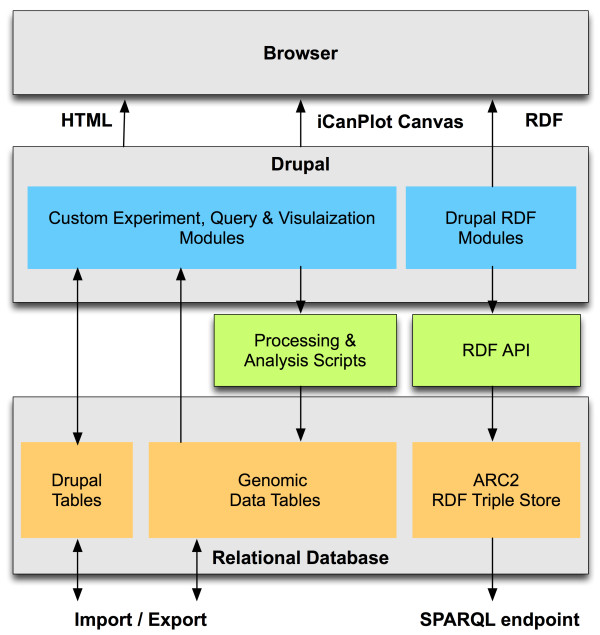
**eXframe architecture**. Custom modules and content types represent the experiment information (metadata) including design, samples and assay information, which are stored in Drupal tables. Molecular data such as gene expression are processed by scripts and stored in the genomic data tables. Drupal RDF modules are used to produce RDF serialization and archived using the ARC2 library.

Our framework, eXframe consists of 1) custom Drupal modules that describe and query the experiment metadata, 2) genomics data tables implemented directly in MySQL that capture the data and annotation associated with genomes, loci, genes, transcripts and probes, 3) processing and analysis scripts, 4) query & visualization tools 5) import and export scripts and finally 6) Resource Description Framework (RDF) modules that produce open Linked Data [20] for the experiments and support the SPARQL [21] semantic query function. The overall architecture of eXframe is shown in Figure 1. The complete framework with all the above components was packaged and made available as a reusable distribution to build repositories of genomics experiments. Installation and configuration of new instances can be done entirely through a web-based interface and does not require programming skills - thus significantly lowering potential barriers to adoption. The next sections describe various components of eXframe in detail. The Semantic Web components - RDF modules, SPARQL endpoint and Linked Data - will be described in detail in a separate article.

### Custom Drupal Modules for Experiment Metadata

We developed several Drupal custom content types to fully describe the experiment metadata. The basic unit of content in Drupal is called a node; nodes are classified by type, and custom modules define new types. The experiment metadata was designed to support multiple types of biomedical experiments and comprises of three primary content types i) Experiment which contains one or more ii) Bioassays that are linked to iii) Biomaterials.

The attributes of an Experiment are title, researcher and the study design details. The Experiment can be linked to publication(s). The Bioassay content type describes the assays performed and has these attributes - type of measurement, technology, platform and the raw output data file produced by the assay. These attributes guide the processing and analysis scripts as well as assist the users to locate their data of interest. The measurement types can be easily extended as new requirements develop. The framework has been designed from the ground up to incorporate new measurement types (such as DNA methylation measurement) or new technologies (such as high throughput qPCR). We capture the technology (such as microarray) as well as the particular platform (such as Affymetrix HG-U133) used in the Bioassay; this enables us to process the raw data in a standard pipeline specific to the type of assay. Bioassays from the same Experiment can be grouped into specific sample and control groups for comparison. We have also developed an intuitive user interface to group Bioassays into the sample and control groups

Bioassays are linked to the Biomaterial content type where sample properties are captured in detail. The default configuration allows the specification of the organism, development stage, tissue and cell types of samples using controlled vocabulary terms. The user can enter the data using either drop down forms or type-ahead fields. Genetic modifications, treatment and disease state of the Biomaterial are also captured as structured annotation where applicable.

We use the Drupal taxonomy system for the controlled vocabulary terms, which are then mapped to various ontologies or taxonomies (Ontologies used and Linked Data generated from experiments will be discussed in a separate paper). The structured annotation of experiments allows enhanced searches - for example a user can find all the data from a particular cell type where histone modification has been measured. Our framework, eXframe, enables a site administrator to customize the set of fields available to the user for annotation. Thus eXframe can be deployed and configured to support new contexts, such as that of clinical data, and important patient characteristics can be acquired in a structured manner.

All the experiment metadata described above can be easily entered into the database using a user-friendly web form (see project website for details). The structured experimental metadata is subsequently processed and made available in several standard formats. This eliminates the need for crafting complex formats by a biologist or curator to generate structured annotation.

### Genomics Data Tables

To enable query by genomic entities and integrate the data, we designed a set of tables that represent the data associated with genomic features such as genes, transcripts and loci as well as their relationships with each other.

The data produced in an experiment is primarily stored in two types of tables. Data from a microarray experiment is stored in a data table (*rtype_data_matrix*) and is associated with a bioassay and a probe. This generic data table can also be used for other technologies which have a data point associated with a probe such as qPCR. Sequencing data, on the other hand, is associated with an arbitrary genomic region with a defined start and end and is stored in the *rtype_locus *table. Computed values such as fold change are stored in the *rtype_fc_matrix *table. The complete genomics database schema is available as Additional File 1.

Genomics data often needs to be described using heterogeneous entities. We designed the database in a flexible manner to accommodate genomics data that is associated with a gene, transcript, probe or genomic interval. For example, each microarray represented in the database has multiple probesets, each probeset may by associated with a transcript, each transcript is associated with a gene. Affymetrix probesets, gene transcripts, etc. are linked to the gene which allows the users to query based on gene symbol and pull the relevant data from different assays. For sequencing assays, resulting values are linked to genomic features, e.g., peaks from a ChIP-Seq assay are linked to the nearest transcript.

Genes may have multiple symbols and orthologs. Orthologous genes are grouped using information from the NCBI HomoloGene database [22] and the homolog id was applied as the group identifier (see Additional File 1). Storing the ortholog information and gene aliases allows the user to query by any gene symbol and retrieve results across species.

### Processing & Analysis Scripts

**Figure 2:**
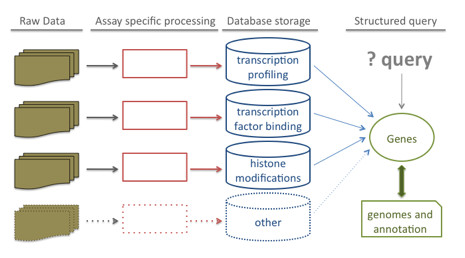
**Genomics data processing and storage**. Raw data produced from various assays are processed according to the assay type; final scores are stored in appropriate database tables and linked to genes, transcripts and other genomic annotations. The database structure enables queries that integrate information across experiments.

We have developed a computational pipeline that allows structured storage, analysis and retrieval of data from different types of platforms (Figure 2). When a user submits the raw data files, the relevant processing is triggered and a job with the appropriate job type is stored, based on the type of assay.

For microarray data, the user uploads CEL files; the data is background-corrected, normalized and summarized using the GCRMA algorithm [23]. Expression fold change between the case and control groups is computed for each probeset and stored in the database along with associated statistics including p-value, false discovery rate, t-statistics, lower & upper confidence intervals, standard deviation (SD) and case and control means. This information enables users without any programming experience to query for a gene fold change across all experiments from various species, disease states and cell types using an easy to use interface. The query results can be filtered by various attributes of the experiment.

Next generation sequencing technologies can be used for measuring RNA expression (RNA-Seq), transcription factor or any protein binding to DNA (ChIP-Seq), histone modification (ChIP-Seq), DNA methylation (RBBS), or protein binding to RNA (RIP-Seq). Users upload FASTQ files for all next generation sequencing assays and the data is consistently processed through the pipeline. The common first step for all next generation sequencing assays is to align the reads to the genome. Subsequent processing and analysis is done depending on the assay/measurement type.

To quantify the histone modification for a gene locus, first reads are aligned using the bowtie program [24] and then the fragments per kilobase per million fragments mapped (FPKM) abundance measure is calculated for the region of interest. For example, the window used was 1Kb upstream to 1Kb downstream of the transcription start site for H3K4me3 and H3K27me3. For transcription factor binding assays, peak identification is done using the MACS program [25] and then peaks are assigned to the gene in whose promoter region it is located. The peak score for each gene is stored in the *rtype_locus *table in the database. For RNA-Seq data, reads are aligned using tophat [26] to identify splice junctions and further processed using cufflinks [27]. The FPKM abundance measure for each transcript is stored in the *rtype_locus *table. The intermediate files - BAM from bowtie/tophat, FPKM from cufflinks and BED/WIGGLE from MACS are also stored for use with other genome browsing tools.

The advantage of assigning all measurements to a gene is that it allows us to compare features (such as DNA methylation, expression, transcription factor binding in promoter region) across experiments using query and visualization tools described in the next section. Further documentation for the pipeline can be found at our project website. Tools for analyzing DNA-methylation and RIP-Seq assays, as well as for SOLiD sequencing platforms are under development and will be available shortly.

### Query & Visualization Tools

We provide various analysis and visualization tools to probe the genomic data and present an integrated platform for genomic discovery. We provide two different forms of visualizations. First, we allow users to query a list of genes and visualize the result as a heatmap illustrating gene expression across all samples (Bioassays) in the Experiment. The second type is a scatter plot of the data - we integrated the iCanPlot tool [28] into eXframe for this purpose. Users can choose the x-axis, y-axis, color and size of the points in the scatter plot from any of the available experiments. Using the scatter plot tool, users can do integrative analysis such as investigating the relationship between histone modifications or transcription factor binding and gene expression.

### Import & Export

The experiment information and genomic data can be downloaded in various formats, including the original raw data file, NCBI GEO SOFT [29], ISA-Tab [14] for the experiment metadata and GCT files for microarray expression data. In future we will also allow download of the processed files, such as the aligned reads (BAM) or peaks (BED/WIGGLE) through the web interface. If researchers enter their data and annotation on the website, they can easily submit the experiment to GEO [1] using the SOFT format, thus providing an incentive for data entry. We also allow import from SOFT files and thus allow users to upload publicly available data from GEO into the database.

## Results

We illustrate the features and benefits of eXframe using two different use cases and present various queries and visualization examples.

### Case study 1: HSCI Blood Genomics

**Figure 3:**
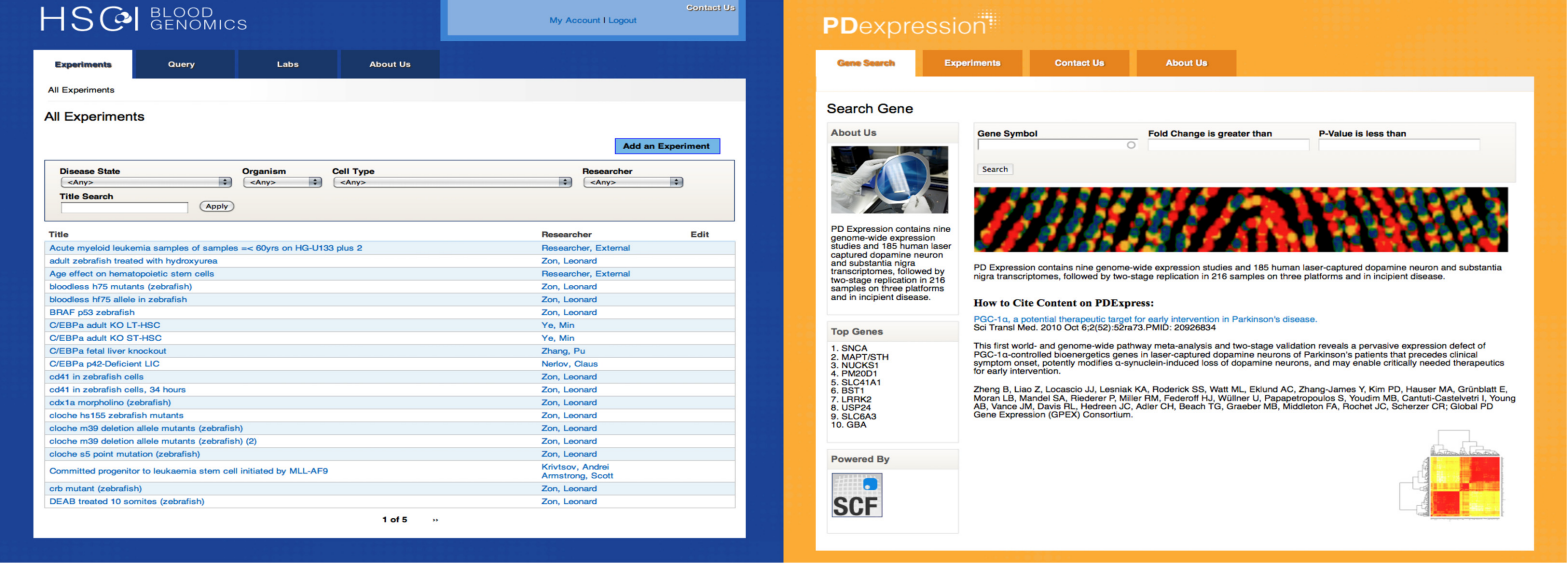
**Screenshots of repositories**. A) HSCI Blood genomics. A repository of over 80 gene expression, epigenetics modification and transcription factor binding experiments performed on hematopoietic stem cells. B) PDExpression. Database of expression studies from Parkinson's disease patients. Site contains data from 185 human laser-captured dopamine neuron and substantia nigra transcriptomes.

The first use case we implemented was a repository for the Harvard Stem Cell Institute (HSCI) Blood program - HSCI Blood Genomics (bloodprogram.hsci.harvard.edu). A screenshot is shown in Figure 3A. The HSCI Blood program focuses on understanding and identifying the molecular and cellular characteristics and pathways involved in the self-renewal of hematopoietic (blood) stem cells. The HSCI Blood Genomics repository is currently comprised of highly curated data from many gene expression, epigenetic modification and transcription factor binding studies using both microarrays and next generation sequencing platforms. There are over 80 experiments with 1000 assays from 3 different organisms, 7 tissue types and 20 cell types. The repository has data from 6 participating laboratories as well as public data that is of interest to the blood program researchers. A subset of the experiments, results of which have been published in scientific journals, is available to the public. The experiment metadata is available to all HSCI members but only lab members can access the raw data. We plan to extend availability of this platform to all laboratories in HSCI over the coming year, with assistance for importing legacy data.

**Figure 4:**
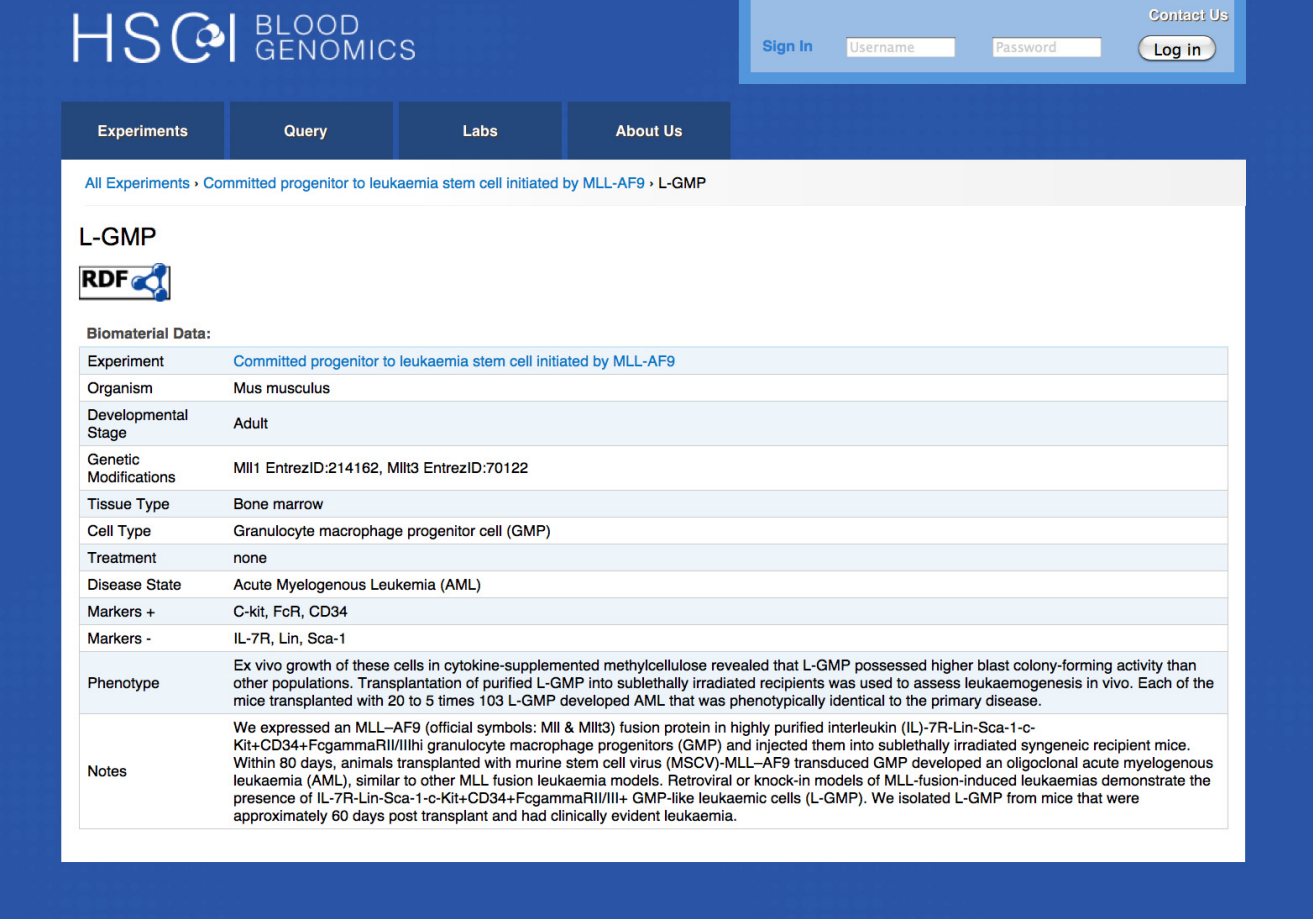
**Structured Annotation of L-GMP samples**. Biomaterials are deeply annotated with structured vocabularies. Sample properties - organism, developmental stage, tissue and cell type are captured. Any genetic modifications, treatment or disease states are also associated with the sample. Free text allows users to enter other important information about the sample.

The biomaterials used in each experiment in the repository have deep and structured annotation. An example of a biomaterial, Granulocyte Macrophage Progenitor like leukemic cells (L-GMP), is shown in Figure 4. The researchers were able to characterize the biomaterial used in the assay using controlled vocabulary terms. There is much debate in the stem cell community about the presence and absence of markers in various hematopoietic stem cell types. Hence, it is important to explicitly state the positive and negative or high/low markers used to sort and isolate the cells. Thus, a separate field is used to specify the antibodies used. For the L-GMP sample, *MLL-AF9 *fusion gene was expressed in mice to create the leukemia model and C-kit+, FcR+, CD34+ and Lin- antibodies were used to isolate the Granulocyte Macrophage Progenitor (GMP) cells from the bone marrow. The identifiers of the genes in the genetically modified L-GMP specimen are also shown in Figure 4. The phenotype or other important notes are captured in a free text format.

The repository contains both data generated at HSCI as well as public data of interest to the community. We downloaded data from the NCBI GEO repository [1] and imported it into the repository using the SOFT format. The data in the repository can be downloaded as various formats including ISA-Tab and SOFT. The format of the resulting ISA-Tab files was independently validated by the ISA-Tab Validator. The repository also makes the data available as a SPARQL endpoint, which will be described in a separate paper.

### Case study 2: PDExpression

**Figure 5:**
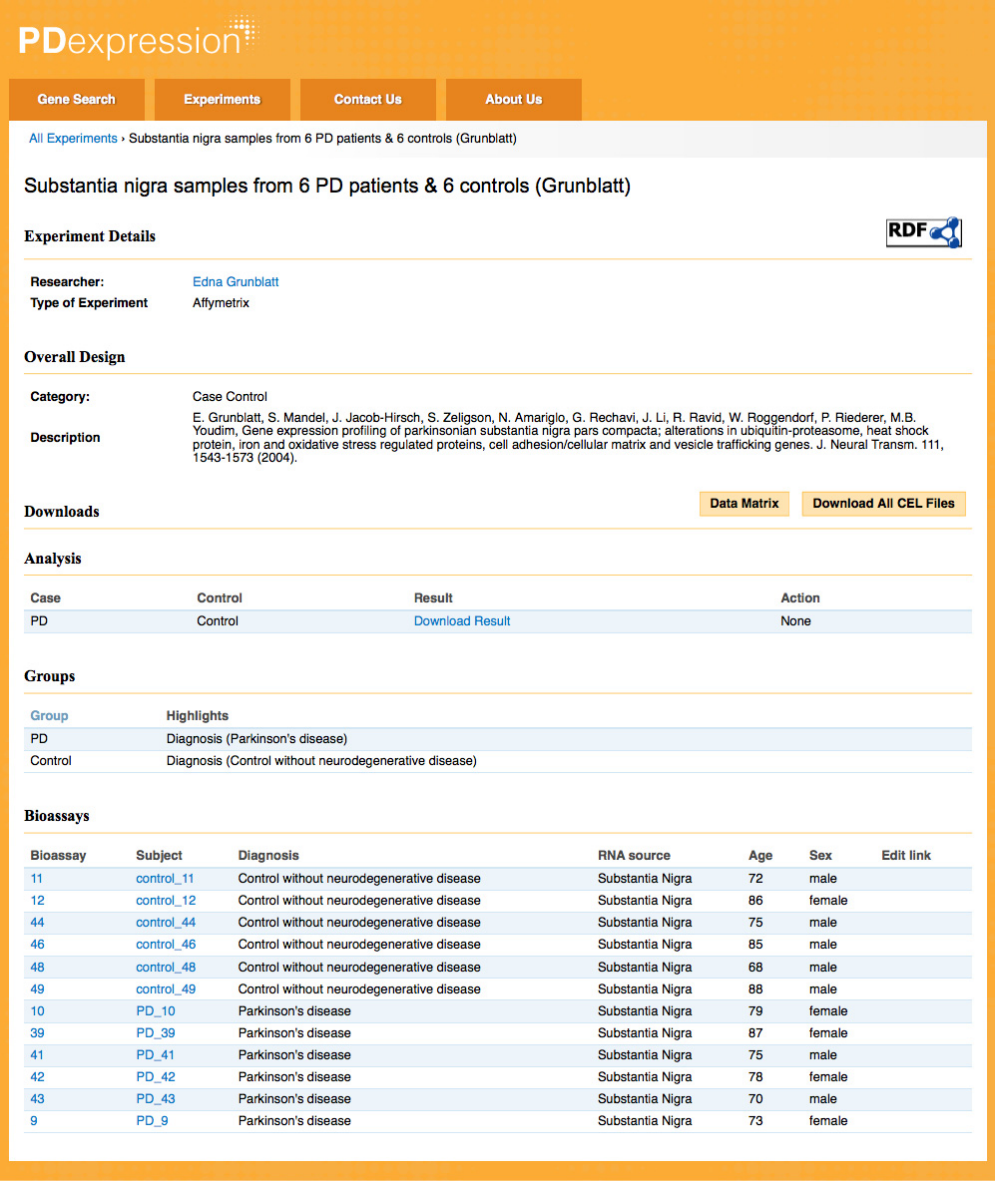
**Sample experiment in PDExpression**. Subject characteristics such as diagnosis and RNA source are captured using controlled vocabularies; age and sex are also noted.

We successfully reused eXframe in a different context to build PDExpression - a repository of transcriptional profiles from Parkinson's disease (PD) patients. PDExpression is still under development and not open to the public. While PDExpression has the same underlying structure as the HSCI Blood Genomics repository, it has a different visual theme that provides a unique and relevant look for this group (Figure 3B). Appearance may be customized as required, by other users of eXframe, using the various "theming" capabilities and modules built in to Drupal. PDExpression currently contains nine genome-wide expression studies from 185 human laser-captured dopamine neuron and substantia nigra transcriptomes using various microarray platforms. In this repository, it was important to capture the patient/subject characteristics and hence the biomaterials were annotated with taxonomy of PD diagnoses and RNA sources; age and sex of the patients were also stated (Figure 5).

### Gene queries

**Figure 6:**
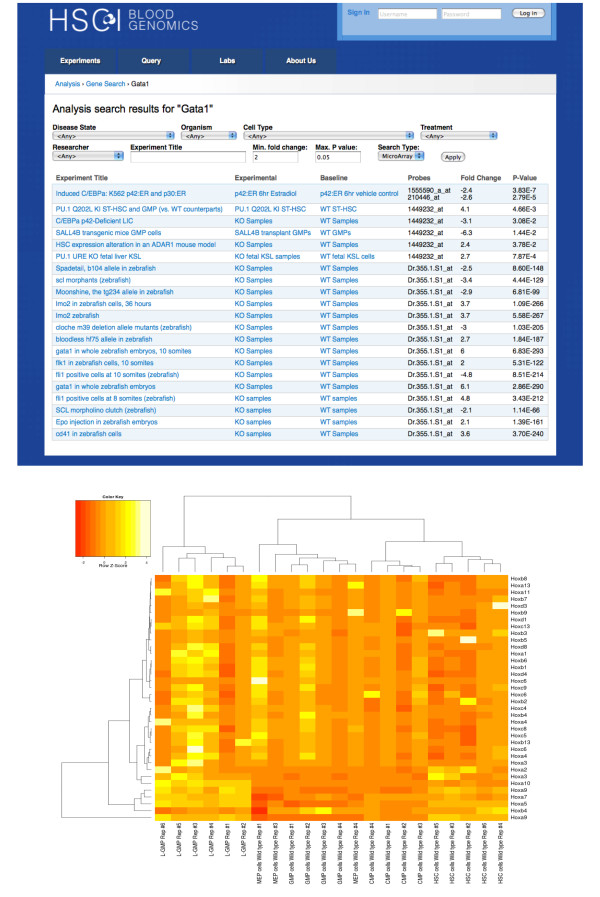
**Gene query and visualization**. A) Query results for GATA1. Results can be filtered using various sample attributes such as organism, cell type or disease state; experiment title or researcher and fold change or p-values. B) Heatmap of HOX gene family. HOX gene expression across various hematopoietic cells - MEP (Megakaryocyte-Erythroid progenitor cell), GMP (Granulocyte-Macrophage Progenitor), CMP (Common Myeloid Progenitor), L-GMP (GMP-like leukemic cells) and HSC (Hematopoietic Stem Cells) - is illustrated.

The structured design of the software and consistent processing and storage of all experiments enable queries by gene symbol. Genes with significant expression changes across different experiments from various cell types, disease states and treatments are returned. The underlying data model, which specifies orthologs, returns significant fold changes of the query gene as well as its orthologs on request. For example, a query for "*GATA1*" currently returns results from human, mouse and zebrafish experiments (Figure 6A). Users are able to narrow down and filter the results using various attributes such as fold change, cell types and organism. The scores for next generation sequencing assays can also be queried in a similar manner.

Researchers are often interested in a family of genes and hence multiple gene queries are also supported. Users can paste a list of genes in a text area and the results are visualized as a heatmap. *HOX *gene family expression in MEP (Megakaryocyte-Erythroid progenitor cell), GMP (Granulocyte-Macrophage Progenitor), CMP (Common Myeloid Progenitor), L-GMP (GMP-like leukemic cells) and HSC (Hematopoietic Stem Cells) cells is illustrated in Figure 6B. The expression values are quantile normalized for the heatmap visualization.

### Next Generation Sequencing - Data Processing and Visualization

To illustrate the processing of next generation sequencing assays, we chose a publicly available RNA-Seq dataset from the NCBI GEO database (GSE30995). In this study, Gabut *et al *investigated the transcriptional effect of alternative splice forms of the *FOXP1 *transcription factor on the H9 embryonic stem cell-line [30]. To study the transcriptional differences of 2 mutually exclusive splice forms of the *FOXP1 *gene, they used custom siRNA pools to knock down (KD) exon 18 and 18b of the *FOXP1 *gene. Control siRNAs were also used and all 3 samples were profiled using RNA-Seq on the Illumina Genome Analyzer platform.

**Figure 7:**
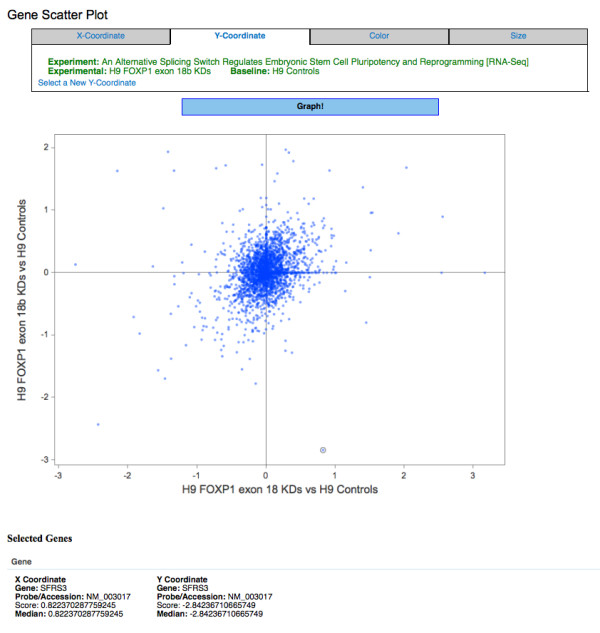
**Scatter plot of sample RNA-Seq data**. The log2 expression fold change of the exon 18b KD vs. control is displayed on the y-axis and exon 18 KD samples vs. control is displayed on the x-axis. Details of the selected SFRS3 gene is shown below the plot.

We first entered the experimental information and annotated the assays and samples using controlled vocabulary terms. Then the sequencing run reads (FASTQ formatted files) were aligned to the human genome assembly hg18 using tophat [26]. The transcript abundance was computed using cufflinks [27] -the FPKM measure for each transcript was stored in the *rtype_locus *table and the fold change vs. control was stored in the *rtype_fc_matrix*. The expression fold change of the exon 18b KD and exon 18 KD samples were plotted using the scatter plot tool (Figure 7). The details of the selected gene with decreased expression in exon 18b KD and increased expression in exon 18 KD is displayed. Such an overview is an easy first step for visual exploration of the entire data. The alignment details can be further explored with any genome-browsing tool that accepts the BAM/SAM format.

### Integrative visualization

**Figure 8:**
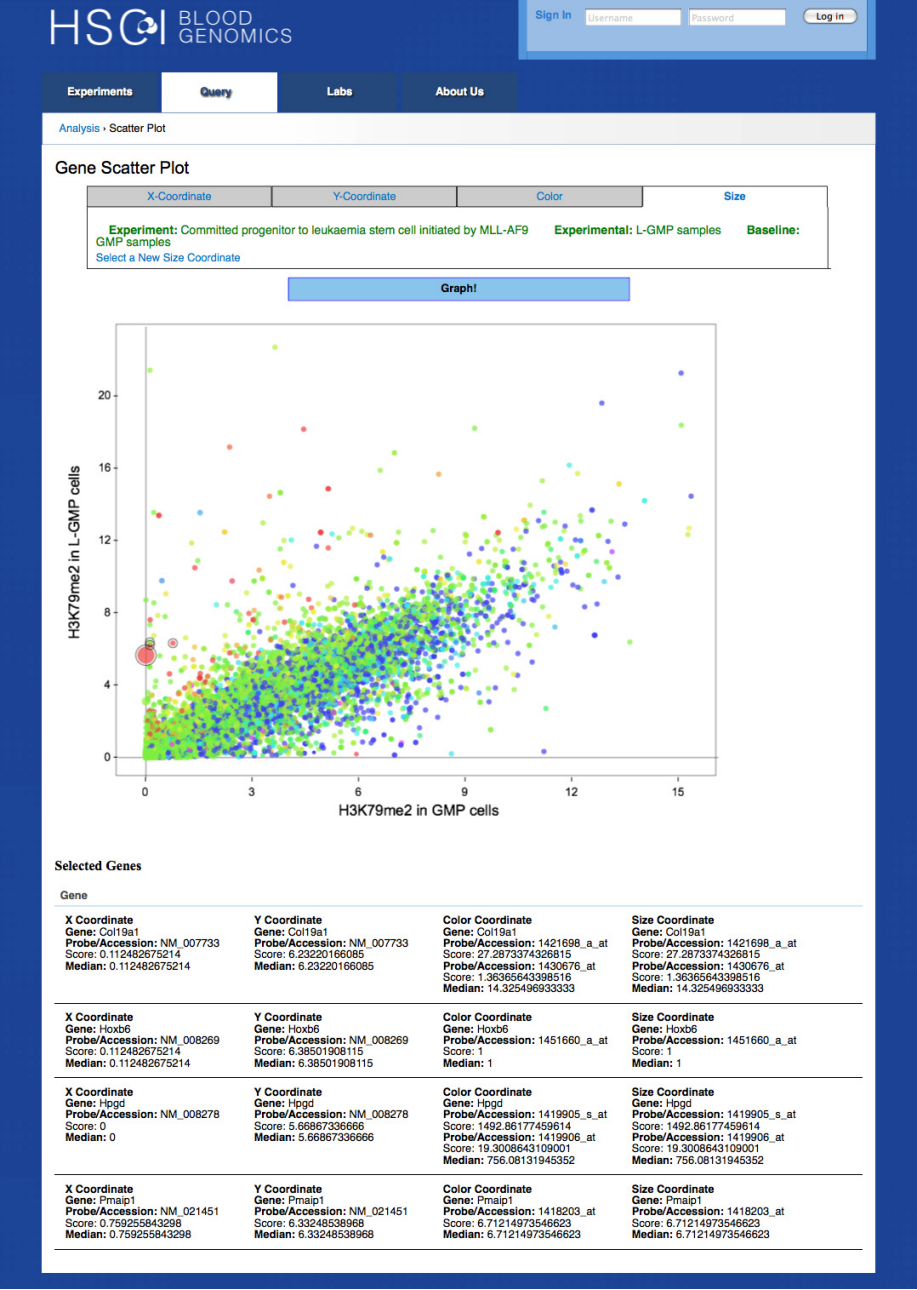
**Scatter plot of histone marks of L-GMP vs GMP cells**. H3K79me2 histone marks of L-GMP and GMP cells are displayed on the y and x-axes. The points are colored and sized by expression fold change of L-GMP vs GMP cells. Details of selected points are shown below the plot.

Further, we were able to successfully use our developed tools to get an integrative view of data generated from different platforms and assays. In this example, we demonstrate the integration of gene expression with histone modification data where gene expression was measured using microarrays and histone modification was measured using ChIP-Seq assays. The histone modification scores were summarized for each transcript and plotted to identify the relationship between different marks in L-GMP and GMP cells. Further, gene expression was superimposed to obtain an integrative view of the role of histone modifications on the gene expression. Figure 8 displays a scatter plot of H3K79me2 histone marks upstream of genes in L-GMP vs GMP cells and the points are colored by expression fold change of L-GMP vs GMP cells. Genes with the highest increase in expression in L-GMP versus GMP (points shown in red) also have the greatest increase in H3K79me2 histone marks in L-GMP compared to the normal GMPs. Such features quickly enable the user to visualize the association of gene expression with histone modification. Users can perform similar visualization for other data types - such as investigating effect of transcription factor binding on gene expression or cross-species concordance.

## Conclusions

We have developed a Drupal-based, reusable, open-source framework - eXframe - that has allowed us to deploy the same software distribution for two widely different use cases and communities. One of them contains transcriptional profiles, histone modifications and transcription factor binding experiments on hematopoietic cells and another on primary tissue derived from Parkinson's disease patients. For both cases, eXframe was used to provide (a) institutional memory of experimental results, (b) cross-dataset comparison, (c) expedited and simplified integration with public databases, and (d) metadata-enabled cross-experiment and cross-laboratory dataset discovery. In the future, other scientific communities or research institutions are encouraged to configure and deploy this highly useful, reusable toolkit for their custom use.

The consistent processing and storage of the experiments enable users to integrate data across labs, species, technologies and measurement types. All data is mapped back to the relevant region of the genome, transcript or gene and thus allows researchers, for example, to investigate the effect of histone modification on the transcription of the gene. It allows cross species or experimental model comparisons. In future, we would like to research document-oriented databases such as MongoDB or implement caching mechanisms to allow scaling for larger data sets.

Structured annotation and use of controlled vocabularies to describe the biological samples, assays and experiment promotes reuse of data. Such an approach allows us to leverage the Semantic Web technologies. Semantic Web produces machine-readable content that allows data reuse and integration with other knowledge resources - eXframe provides the ability to generate Linked Data and SPARQL endpoints for the experimental metadata. The easy to adopt system lowers the barrier of entry and provides the benefits of the Semantic Web, while effectively hiding the complexities of the technology. These features will be fully described in a forthcoming paper.

Open-access, standardized annotation allowing interoperability and analysis ready data repositories are required for integrative genomics [31]. We believe that use of our framework will encourage data sharing, integration and meta-analysis of genomics data, which will ultimately lead to the understanding of complex biological processes and pathogenesis of diseases. This toolkit supports, we believe, a broader and more comprehensive feature set than any other genomics experiment repository code available for general re-use under open source license. We encourage both use and collaborative extension of eXframe by other researchers and informaticians.

## Availability & Requirements

• Project Name: eXframe

• Project Home page: http://sciencecollaboration.org/exframe

• Operating System: Platform independent

• Programming Language: PHP & R

• Other requirements: LAMP stack

• Availability: freely available under a GNU 2.0 license without any restrictions for commercial use The web application is supported on the following browsers - Firefox 4, Safari 5, Chrome 10, IE 9 or higher.

## Authors' contributions

AS and EM were the primary contributors to design and development of software under the guidance and supervision of SD. TC provided consulting on design, functional specifications and open source, reusable software development. Sample data described in the paper was generated in SA's laboratory. Manuscript was authored by AS & SD with contributions from TC & EM. All authors read and approved the final manuscript.

## Supplementary Material

Additional file 1 **Genomics Tables**. Database schema of the genomics tables Click here for file
