# Supplementary figures and images for: eXframe: reusable framework for storage, analysis and visualization of genomics experiments
Source: BMC Bioinformatics. 2011 Nov 21;12:452. doi: 10.1186/1471-2105-12-452 (PMC3235155; doi:10.1186/1471-2105-12-452)

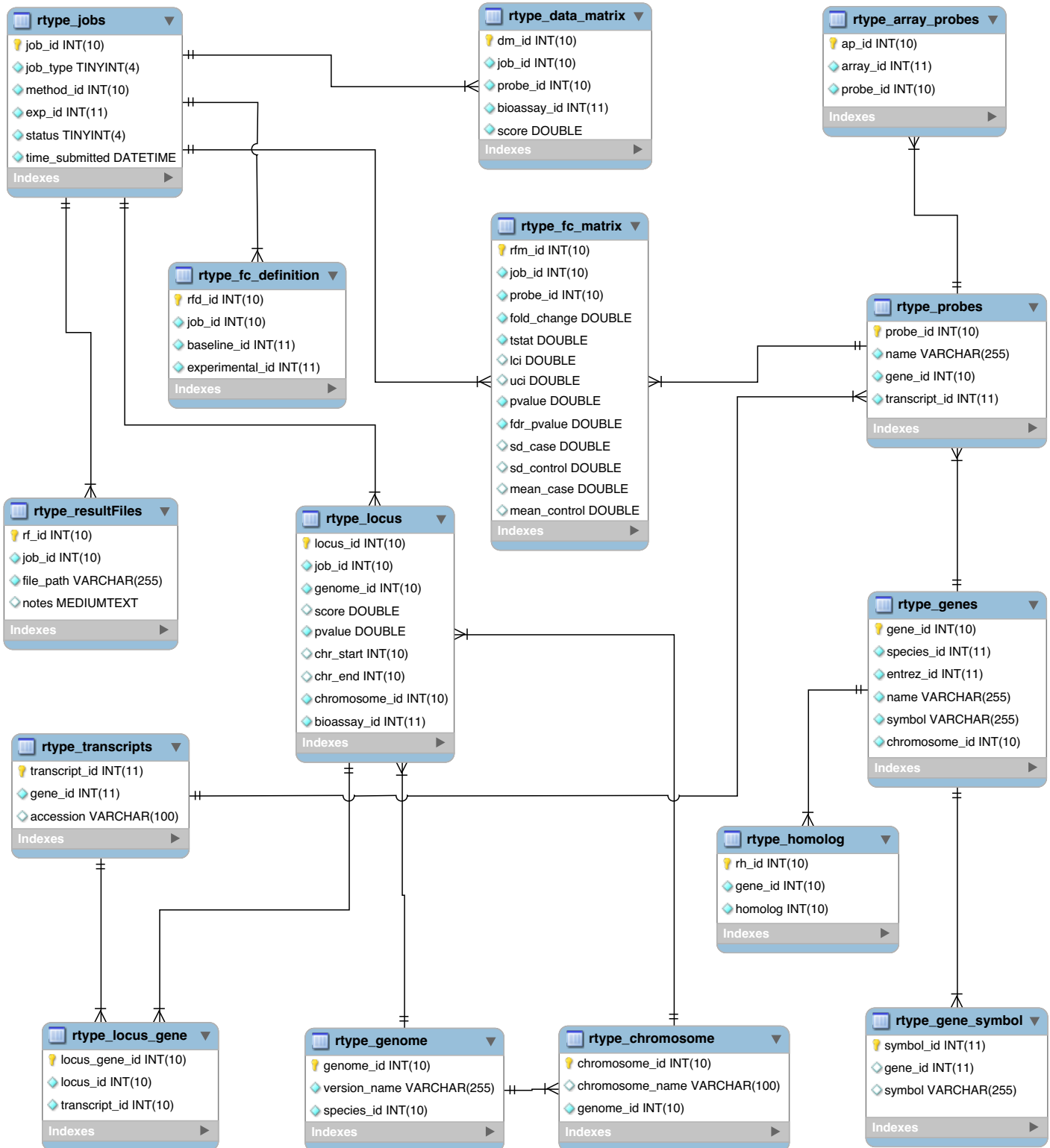

Supplement: Additional file 1 — Genomics Tables. Database schema of the genomics tables [file 1471-2105-12-452-S1.PDF]
